# Impact of Environmental and Anthropogenic Factors on Mandrill (*Mandrillus sphinx*) Occupancy and Habitat Use in Monte Alén National Park, Equatorial Guinea

**DOI:** 10.1002/ajp.70125

**Published:** 2026-02-17

**Authors:** Tania Guzmán‐Santillán, Salvador Mandujano, Edward Wright, David Fernández, Juan‐Cruz Ondo Nze Avomo, Fidel Esono Mba Eyono, Timothy Bray

**Affiliations:** ^1^ Department of Health Sciences, Bristol Veterinary School, MSc Global Wildlife Health and Conservation Program University of Bristol Bristol UK; ^2^ Red de Biología y Conservación de Vertebrados Instituto de Ecología A.C. Xalapa Veracruz México; ^3^ The Biodiversity Consultancy Cambridge Cambridgeshire UK; ^4^ Department of Anthropology and Archaeology University of Calgary Calgary Alberta Canada; ^5^ Equatorial Guinea Conservation Project Bristol Zoological Society Bristol UK; ^6^ Instituto Nacional de Desarrollo Forestal y Gestión del Sistema de Áreas Protegidas (INDEFOR‐AP) Malabo Equatorial Guinea

**Keywords:** camera traps, habitat use, hunting pressure, occupancy modeling, primate

## Abstract

Understanding the ecological and anthropogenic factors shaping primate distribution is critical for effective conservation, particularly for species threatened by habitat loss and hunting. This study investigates factors influencing mandrill (*Mandrillus sphinx*) occupancy in Monte Alén National Park (MANP), Equatorial Guinea, a protected area with diverse habitats. Using 35 camera traps over 10,858 trap‐days, we recorded 79 mandrill detections (48 in the wet season, 31 in the dry season) and estimated naïve occupancy at 24%. We applied single‐species, single‐season occupancy models to evaluate the effects of environmental and anthropogenic factors, including proximity to water bodies, hunting camps, villages, park boundaries, and terrain slope. Occupancy probability decreased with distance from water, indicating dependence on riparian habitats, while slope had no major effect. Proximity to hunting camps also influenced mandrill distribution, with higher occupancy observed farther from these areas. Seasonal analysis suggested that mandrills concentrated in resource‐rich areas during the wet season, highlighting flexible, site‐specific habitat use. These results emphasize the combined influence of environmental resources and human pressures on mandrill space use. Effective conservation should focus on protecting critical habitats near water sources and regulating human activity around hunting areas. More broadly, our findings improve understanding of how ecological, anthropogenic, and socioeconomic factors shape primate distribution, offering insights relevant for the conservation of other vulnerable species across Central Africa.

## Summary

- Mandrill occupancy in Monte Alén National Park was significantly higher near to water sources, highlighting the ecological importance of riparian zones.
- Mandrills were more likely to occupy areas farther from hunting camps, suggesting avoidance behavior in response to hunting pressure.
- No significant seasonal variation in occupancy was detected, likely due to the region's consistently high rainfall and year‐round fruit availability.
- Mandrills exhibited a patchy distribution across the park, with most detections in the northeast, suggesting localized environmental or anthropogenic influences.

## Graphical abstract

Camera trap data showed that mandrills were not found evenly distributed across MANP. Mandrill occupancy decreased when distance to water bodies increased. Whereas, when distance to hunting camps increased, mandrill occupancy decreased.

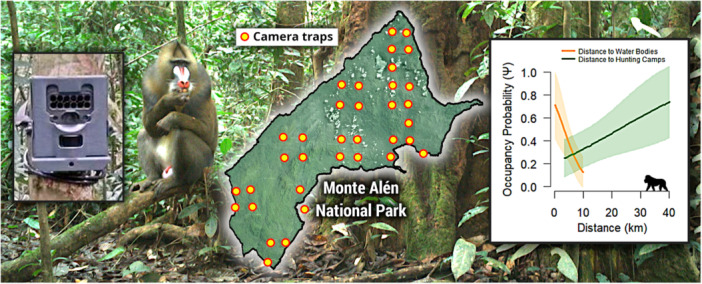

**Abbreviations**
AIC: Akaike Information Criterion
CFA: West African CFA franc
MANP: Monte Alén National Park
SD: standard deviation
SE: standard error

## 1 Introduction

Habitat use and selection are fundamental ecological concepts that explain how animals distribute themselves across landscapes to maximize survival and reproductive success (Hamilton 2019; Mayor et al. 2009). These processes are shaped by resource availability, predation risk, competition, and abiotic factors such as topography, climate, and seasonality (Gautier‐Hion et al. 1981; Grand 2002; Morris 2009; Sousa et al. 2014). In addition to these natural drivers, human activities strongly influence habitat use. In human‐modified landscapes, fragmentation and connectivity play particularly important roles (Arroyo‐Rodríguez and Mandujano 2009). Fragmentation reduces habitat continuity, restricts movement, and alters ecological processes, whereas connectivity promotes gene flow and resource access, mitigating some negative effects (Cushman et al. 2013; Mullu 2016; Rudnick et al. 2012). Among anthropogenic disturbances, hunting is especially pervasive, causing not only direct mortality but also behavioral shifts, such as spatial avoidance, altered activity patterns, and reduced group sizes (Croes et al. 2007; Laursen et al. 2016; Mendes et al. 2020; Remis and Jost Robinson 2012). These responses shrink the range of habitat perceived as safe, effectively fragmenting the landscape from the animal's perspective (Mendes et al. 2020).

Studying species' space use and occupancy is therefore critical for understanding ecological processes and informing conservation (Farris et al. 2019; Guillera‐Arroita et al. 2010; Nichols et al. 2008). However, monitoring mammals in tropical forests is notoriously difficult, particularly primates that occur at low densities, occupy large home ranges, and often modify their behavior in response to human presence (Buckland et al. 2010; Hongo 2023; Plumptre 2000). Advances in non‐invasive tools such as camera traps now allow researchers to assess species presence and habitat use with minimal disturbance (Boyer‐Ontl and Pruetz 2014; Caravaggi et al. 2017; Fournier et al. 2023). To account for imperfect detection, where species present at a site may go undetected, occupancy modeling provides a framework to link detection histories with environmental and anthropogenic factors (MacKenzie et al. 2002, 2017), and has been successfully applied to primates to reveal distribution patterns and threats (Baker et al. 2011).

Equatorial Guinea, ranked fourth in Africa for primate species richness, forms part of a major sub‐Saharan hotspot (Brooks et al. 2006; Chapman et al. 1999). Monte Alén National Park (MANP) harbors 16 primate species, including the mandrill (*Mandrillus sphinx*), listed as Vulnerable by the IUCN (Abernethy and Maisels 2019). These terrestrial primates live in multi‐male, multi‐female groups up to > 800 individuals (Abernethy et al. 2002; Hongo 2014), occupying expansive home ranges of approximately 50 km² (Jouventin 1975), though recent studies report much larger ranges up to ~74 km² (White et al. 2010). As omnivorous seed dispersers and indicators of forest health, they play key ecological roles and contribute significantly to primate biodiversity in Central Africa (Harrison 1988; Hladik and Hladik 1967; Lahm 1986).

Historical records indicate that in 1997, mandrills were widely distributed across MANP and observed in all surveyed areas, despite being commonly hunted at the time (Garcia and Mba 1997). More recent studies suggest intensified hunting pressure, with densities estimated at approximately 41 individuals/km², although confidence intervals were wide because of limited observations (Kümpel et al. 2008; Rist et al. 2009). Mandrills are among the most heavily targeted primates for wild meat, with pursuit probabilities exceeding 90% when encountered by hunters, and juveniles disproportionately affected (Kümpel 2006; Kümpel et al. 2008). While time‐series data are limited, these records indicate that intensified hunting pressure over recent decades has likely contributed to local population declines (Kümpel et al. 2008).

These pressures are compounded by growing demand for wild meat, driven by population growth and improved hunting technology (Fa et al. 2005; McNamara et al. 2016). In rural areas lacking alternative protein sources, wild meat remains essential for food and income (Fa et al. 2005; Ingram et al. 2021; Nasi et al. 2011), providing 30–180 g of protein per person per day in the Congo Basin (Fa et al. 2003). In Sendje, a village in Equatorial Guinea, hunting was the main income source, with average monthly earnings of 36,650 CFA (Central African CFA franc; ~ $66 USD) and top hunters earning up to 195,678 CFA per month (~$352 USD) (Kümpel et al. 2010a). Similarly, in north‐east Río Muni villages on the mainland of Equatorial Guinea, hunting persists where agriculture is less profitable, mainly benefiting middle‐income households, while poorer ones often lack adult males to hunt (Kümpel et al. 2010a). Hunting has also driven primate declines across the region. On Bioko, the Bioko red colobus (*Procolobus pennantii*) has vanished from much of its range (Butynski and Koster 1994; Cronin et al. 2016), and throughout Central Africa, intensive hunting has reduced primate populations, sometimes leading to local extinctions (Cronin et al. 2016; Magnuson 2005; Rovero et al. 2012; Wilkie et al. 2005; Ziegler et al. 2016). Even near protected areas, such as MANP, skeletal remains in villages indicate that mandrills remain vulnerable (Rosas et al. 2021).

MANP, with its diverse vegetation, river networks, and varied topography, provides environmental conditions that strongly influence mandrill habitat use. Its proximity to nearby villages and the city of Bata, a known hub for wild meat trade, exposes mandrills to hunting pressure that may affect their occupancy. Despite their ecological and conservation importance, mandrill populations in MANP remain poorly studied, and knowledge gaps in their distribution and ecology have been identified as priorities in the *Cercocebus* and *Mandrillus* Conservation Action Plan (2024–2028), prepared by the IUCN SSC Primate Specialist Group to guide global conservation efforts for these taxa (Dempsey et al. 2024). Addressing these gaps is critical for understanding how ecological and anthropogenic factors shape mandrill space use in one of their last strongholds.

In this study, we used a single‐species, single‐season occupancy modeling framework (MacKenzie et al. 2017) to investigate mandrill spatial ecology in MANP. Based on habitat selection theory and the expected effects of human disturbance and environmental heterogeneity, we predicted that seasonality and topography would influence resource use and movement, while proximity to hunting camps would reduce occupancy, and proximity to water sources would increase occupancy. Specifically, our objectives were to: (1) assess site‐specific differences in mandrill detection; (2) evaluate the influence of hunting camps on mandrill occupancy; and (3) examine how environmental and anthropogenic factors influence occupancy and seasonal patterns of habitat use.

## 2 Materials and Methods

### 2.1 Ethics Statement

All research protocols were reviewed and approved by the Bristol Zoological Society ethics panel, approval number BZS_2023_049. The study was conducted in accordance with legal regulations for animal research in Equatorial Guinea. Fieldwork was carried out under a general agreement by the Forestry department of Equatorial Guinea (INDEFOR) without the requirement for specific permits, as granted by Director Fidel Esono Mba. This research also complied with the American Society of Primatologists (ASP) Principles for the Ethical Treatment of Non‐Human Primates and followed the ASP Code of Best Practices for Field Primatology.

### 2.2 Study Site and Species

**FIGURE 1:**
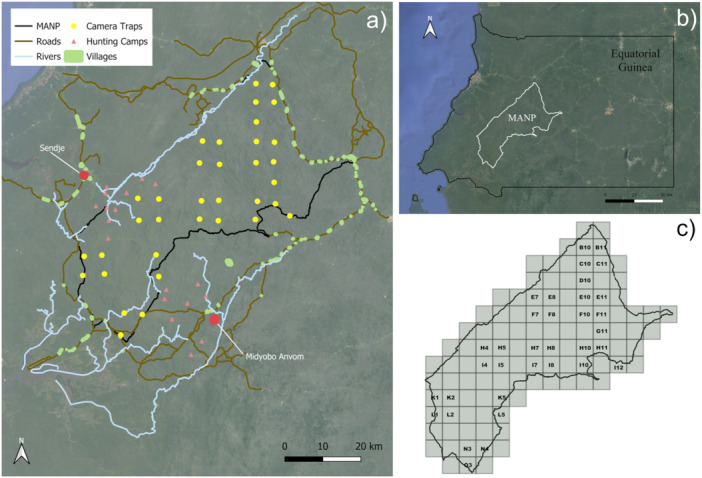
Overview of Monte Alén National Park (MANP), Equatorial Guinea. (a) Map of the park showing the locations of 35 camera traps (yellow circles), 19 hunting camps (red triangles), surrounding villages (green polygons), and main rivers (blue lines). (b) Location of MANP within Equatorial Guinea. (c) Map of the park divided into grid cells, with each grid cell labeled with the name of the camera trap located within it. Maps were created in QGIS v3.34 (QGIS Development Team 2024).

This study was conducted in MANP, a ~ 200,000‐hectare reserve characterized by rugged topography, with elevations ranging from 400 to 1000 m above sea level (Garcia and Mba 1997). Several rivers traverse the park's northern, central, and southern regions, and roads connect surrounding villages, many of which lie along the park's perimeter, creating a dense human presence (Figure 1a). MANP is located in Equatorial Guinea (1.5000° N, 10.2500° E; Figure 1b) and includes a variety of ecosystems, such as lowland rainforest, mountainous rainforest, secondary forest, savannah, and grassland (Garcia and Mba 1997; Sunderland et al. 2006). The park supports a high level of biodiversity, hosting 246 bird species, 27 amphibian species, 25 reptile species, numerous plant species, and at least 35 mammal species (Kenfack 2013), including the mandrill. The region experiences two wet seasons (March–May and September–November) and two dry seasons annually (Kümpel et al. 2008). Annual rainfall ranges from 2000 to 3000 mm (Fa 1991), and temperatures average at a minimum of 25ºC throughout the year (Kümpel 2006).

### 2.3 Anthropogenic Context: Nearby Villages and Hunting Camps

Nearby villages and hunting camps are described to provide context for evaluating anthropogenic effects on mandrill occupancy. Although several villages surround MANP, Sendje and Midyobo Anvom are highlighted here because they are the two villages known to supply wild meat to Bata (Kümpel 2006; Kümpel et al. 2010b; Rist et al. 2009). While the most recent quantitative data are somewhat dated for Sendje (2002–2004; Kümpel 2006; Kümpel et al. 2010b) and Midyobo Anvom (2005–2006; Rist et al. 2009), they provide valuable insights into hunting structure and intensity in this remote region.

In Sendje, at least 10 active camps were recorded between 2002 and 2004 (Figure 1a; Kümpel 2006; Kümpel et al. 2010b). Each camp hosted 5–6 hunters for 6–9 months before rotating, indicating near year‐round hunting. Hunting was primarily with wire snares, with shotgun use increasing over time. Offtake studies documented over 9000 animals from 49 species, including mandrills, harvested at unsustainably rates (Kümpel 2006). In Midyobo Anvom, eight hunting camps were active during 2005–2006 (Figure 1a; Rist et al. 2009). Hunting followed a rotational system, with 50–80 hunters active at any time, approximately 60% in camps and the remainder from surrounding villages. Camps were partially occupied, with hunters rotating according to prey availability and returning after months or years. Methods included wire snares, occasional firearms, and hand or lasso capture. Hunting occurred year‐round and targeted mandrills alongside other primates and non‐primate prey (Rist 2008). The legal status of these camps is unreported, so it is unclear whether they were officially sanctioned.

### 2.4 Camera Trap Deployment and Detection Metrics

To evaluate spatial variation in mandrill occupancy and habitat use, the Equatorial Guinea Conservation Program of the Bristol Zoological Society deployed 35 camera traps across throughout MANP. We divided the park into 111 grid cells, each measuring 5 × 5 km² (Figure 1c). Although two grid cells (L1 and L5) lay outside the official park boundaries, we included them because forest habitat extends beyond the park, and previous research found no difference in species diversity between protected and adjacent unprotected areas (Murai et al. 2013). From the 111 available grid cells, we selected 35 using stratified random cluster sampling, defining strata to exclude areas near known game trails to minimize behavioral bias associated with proximity to established paths.

We used four camera trap models: Browning Spec Ops, Reconyx, Browning Dark Ops, and Browning Strike Force. We placed each camera at the center of the selected grid cell and mounted it on tree trunk above ground level to optimize detection of terrestrial mammals. We did not use bait to avoid influencing animal movement or behavior. We equipped all cameras with infrared technology to capture nighttime images without disturbing wildlife. Although we installed cameras over 5 years (2019–2024), we analyzed only the data collected between May 2022 and May 2023, when all cameras were consistently operational. Given the 5‐month battery life and autonomous functioning of the cameras, we visited the field every 5 months to retrieve data and minimize disturbance.

We uploaded the camera trap data to the Wildlife Insights website (Wildlife Insights n.d.), which is used by our partner organization and many other organizations worldwide to manage camera trap wildlife monitoring data. The platform also allows data sharing, increasing accessibility and collaboration. Although the platform uses an artificial intelligence system to automatically classify species in the images, we manually reviewed all images to verify the presence of mandrills. For analysis, we treated each camera trap as an individual site. While the 35 camera traps remained active throughout the survey period, some experienced intermittent technical malfunctions. These inactive periods were recorded as missing data (NAs) and were accounted for when calculating a standardized, descriptive measure of mandrill activity. We calculated the photographic rate for each camera station as the number of independent mandrill detection events per 100 camera‐trap days = (Number of events/Active days) x 100 (O'Brien 2011). Station‐level photographic rates were used alongside naïve occupancy estimates to describe spatial variation in mandrill detections across the park.

To capture seasonal variation in occupancy and site‐level habitat use, we constructed separate detection histories for each season, resulting in four detection matrices. We limited each detection history to a maximum of 45 sampling days and collapsed it into five survey occasions, each comprising 9 consecutive days. This structure satisfied the closure assumption required for occupancy modeling (MacKenzie et al. 2017). We then combined the seasonal detection matrices into a single stacked dataset, comprising 252 site‐season combinations and five survey occasions per site.

### 2.5 Environmental and Anthropogenic Covariates

To evaluate environmental and anthropogenic drivers of mandrill occupancy, we identified eight covariates for model construction: five site‐specific variables for occupancy and three observation‐specific covariates for detection. We treated site‐specific covariates as constant throughout the study period, including the distance from each camera trap to the nearest hunting camp, water body, village, and park boundary, as well as terrain slope. We measured distances in QGIS (Quantum Geographic Information System) using the “*v.distance*” tool (see Methods 2.3 for details on hunting camps and nearby villages). We identified village, water body, and park boundary locations using Google Earth Pro, which we found reliable given the high visibility of geographic features. When measuring distances to villages, we included all villages visible in Figure 1, including Sendje and Midyobo Anvom.

Because of limited spatial data, we included only the main rivers in the study area, as comprehensive data for smaller rivers and streams were unavailable. Rivers were included because they likely influence mandrill occupancy by providing essential water resources for drinking and thermoregulation (Lahm 1986; Thompson and Hermann 2024), facilitating foraging in riparian habitats (Lahm 1986), and occasionally providing aquatic prey, such as fish or crustaceans (Jouventin 1975; Owens et al. 2015). Depending on river size and season, rivers may act as barriers, restricting mandrill movement and dispersal and thereby shaping patterns of habitat use (Pavelka et al. 2025). We estimated slope in Google Earth Pro, based on elevation changes surrounding each camera trap location.

For observation‐specific covariates, which varied throughout the study period, we included season, sampling effort, and camera model. We categorized season as wet or dry for each survey occasion, reflecting the park's two wet and two dry seasons. We defined sampling effort as the number of days on which each camera trap operated during the 45‐day period used to construct each detection history. We included camera model to account for potential variation in detection performance across the four models deployed in the field.

### 2.6 Occupancy Modeling and Statistical Analysis

We applied a single‐species, single‐season occupancy model to assess how environmental and anthropogenic covariates influence mandrill occupancy patterns and seasonal habitat use within MANP. Although data collection spanned multiple seasons over the course of a year, we treated each season independently and built separate detection histories for the wet and dry seasons. This approach allowed us to assume demographic closure within each 45‐day sampling window, thereby satisfying the assumptions of single‐season occupancy modeling. We did not use a multi‐season model, as our primary objective was not to estimate colonization or extinction dynamics between seasons, but rather to evaluate how environmental and anthropogenic covariates influenced mandrill occupancy within individual seasons.

This modeling framework estimates two key parameters: detection probability (p) and occupancy probability (ψ). Detection probability is the likelihood of detecting a species at a site given where it is present, and it follows a Bernoulli distribution (MacKenzie et al. 2017). In contrast, occupancy (ψ) represents the probability that a species is present at a given site, regardless of whether it was detected during surveys (MacKenzie et al. 2002). We fitted detection and occupancy models using the *occu* function from the *unmarked* package (Fiske and Chandler 2011). Continuous covariates were standardized (mean = 0, SD = 1) prior to model fitting to improve model convergence and allow comparison of effect sizes; predicted relationships shown in figures are presented using covariates on their original (unstandardized) scale.

We used a two‐step model selection procedure based on Akaike's Information Criterion (AIC). First, candidate detection models were specified as: p(.), p(Season), p(Sampling effort), and p(Camera model), where p(.) denotes a null model with constant detection probability. Second, using the selected detection structure, we evaluated candidate occupancy models. Candidate occupancy covariates included: ψ(Distance to water body), ψ(Distance to hunting camps), ψ(Distance to villages), ψ(Distance to park boundary), and ψ(Slope). For both detection and occupancy, all additive combinations and ecologically plausible interactions (e.g., season x sampling effort for detection and distance to village x slope for occupancy) were considered. Candidate models were compared using AIC, and models with low support (ΔAIC > 2 relative to the model with the lowest AIC; see Burnham and Anderson 2002) were not carried forward. When multiple candidate models had ΔAIC ≤ 2, selection among them was guided by predefined criteria: covariates consistently appearing across top‐ranked models were prioritized, and interaction terms were retained only when they substantially improved model fit. Model selection relied entirely on AIC; *p*‐values were not used for variable selection. Effect sizes and 95% confidence intervals were used to interpret model outcomes and to account for model selection uncertainty.

We evaluated model fit with the MacKenzie and Bailey goodness‐of‐fit test (MacKenzie and Bailey 2004) using the mb.gof.test function. The R Script of the complete analysis is provided in Appendix S1, Supporting Information.

To explore potential seasonal shifts in habitat use within MANP, we conducted site‐level occupancy analyses separately for wet and dry seasons. We extracted predicted ψ values for camera sites present in both seasons and assessed seasonal differences with paired *t*‐tests. We also calculated the variance of ψ across sites in each season, with higher variance indicating uneven site use and lower variance reflecting more even distribution. This approach follows the conceptual framework of Hongo et al. (2018), who examined seasonal variation in habitat use using capture rates, but we applied it to ψ to account for imperfect detection. Finally, we visualized occupancy patterns spatially using GIS‐based maps of site‐level ψ values.

## 3 Results

### 3.1 Camera Trap Effort

Camera traps were deployed at 35 stations, each operating for varied durations. The number of active days per station ranged from 144 to 365, with a mean of 310 days (SD = 72.95). Although most stations operated for more than 300 days, a few recorded shorter durations as a result of equipment failure. Based on the collapsing of independent data into successive 9‐day intervals, a total of 79 mandrill detections were recorded (48 in the wet season and 31 in the dry season), over a sampling effort of 10,858 camera‐trap days.

Naïve occupancy was 24%, with higher detection rates during the wet season. Photographic rates ranged from 0.0 to 2.19 events per 100 camera‐trap days (mean = 0.68, median = 0.55, SD = 0.68). Rates were generally higher in the northeastern region of the park, which is largely free of hunting camps, and the lowest in the southern region, where hunting is concentrated. These patterns highlight marked spatial variation in mandrill activity across MANP.

### 3.2 Occupancy Patterns and Covariate Effects

All candidate models with ΔAIC ≤ 2 are presented in Table 1.

**TABLE 1:** Top models for mandrill single‐season, single‐species occupancy and detection probabilities (p = detection, ψ = occupancy).

Model	K	AIC	ΔAIC	AIC weight	GOF	Detection (SD)	Occupancy (SD)
Detection probability							
p(Season) ψ (.)	3	557.00	0.00	0.29	1.74	0.18 ± 0.04	0.47 ± 0.08
p(.) ψ(.)	2	557.07	0.08	0.28	1.59	0.16 ± 0.03	0.45 ± 0.08
p(Effort) ψ(.)	3	558.44	1.45	0.14	1.39	0.18 ± 0.04	0.45 ± 0.08
p(Season + Effort) ψ(.)	4	558.46	1.46	0.14	1.32	0.63 ± 0.08	0.46 ± 0.08
p(Season * Effort) ψ(.)	5	558.60	1.61	0.13	1.29	0.13 ± 0.04	0.46 ± 0.08
Occupancy probability							
p(Season + Effort) ψ(Dis_WB + Slope + Dis_HS)	7	550.00	0.00	0.70	0.96	0.18 ± 0.04	0.40 ± 0.12

Abbreviations: AIC, Akaike Information Criterion; AIC weight, relative likelihood of the model compared to others; ΔAIC, difference in Akaike Information Criterion (compared to the top model); Dis_HS, distance to hunting camps; Dis_WB, distance to water bodies; GOF, goodness of fit; K, number of parameters in the model; p, detection probability; SD, standard deviation; ψ, occupancy probability.

The overall detection probability across the park was 0.18 (SE = 0.04; Table 1). Model selection supported multiple competing detection models, with five candidate models falling within ΔAIC ≤ 2 of the best‐supported model (Table 1). The top‐ranked detection model included both season and sampling effort as covariates, although models, including only season, only sampling effort, or their interaction, received similar support. While detections were higher during the wet season (48) than the dry season (31), the effect of season on detection probability was weak (*β* = 0.42, SE = 0.30, *p* = 0.16), suggesting limited influence at the park‐wide scale. Camera model was evaluated as a detection covariate but not supported in the top‐ranked models based on AIC and is therefore not discussed further.

**FIGURE 2:**
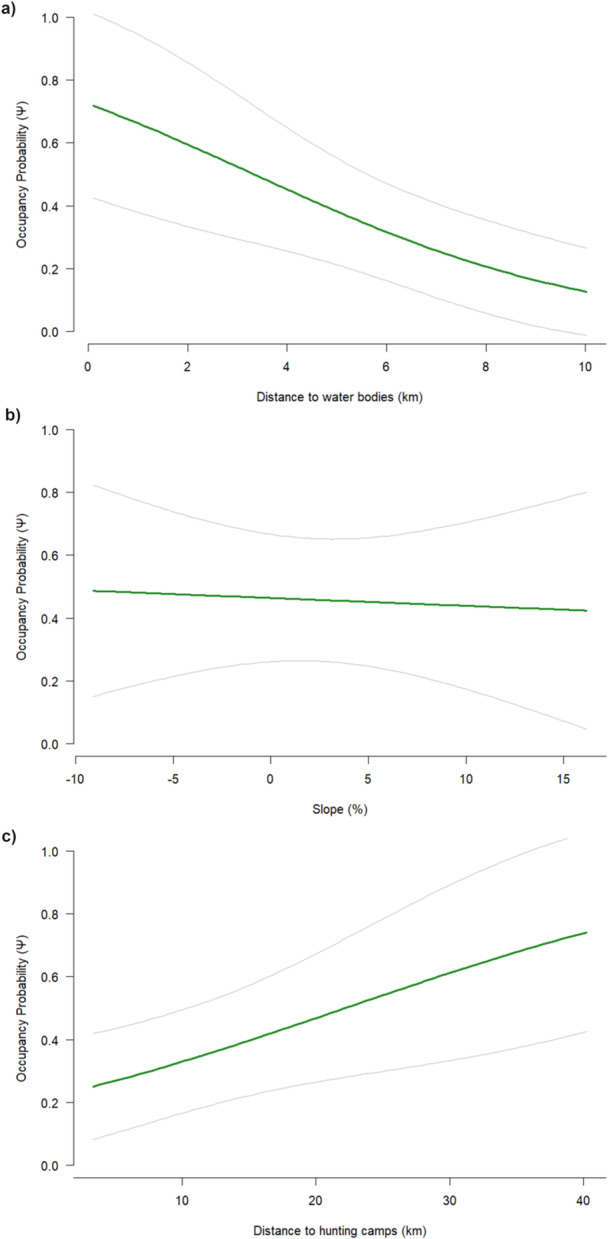
Occupancy probability (ψ) of mandrills with 95% confidence intervals from May 2022 to May 2023 in relation to: (a) distance to water bodies, (b) slope (°), and (c) distance to hunting camps. All covariates are presented in their original scale (not standardized).

In contrast, occupancy model selection supported a single top‐ranked model (ΔAIC = 0; Table 1), with an estimated overall occupancy probability of 0.40 (SE = 0.12). Distance to villages and distance to park boundaries were evaluated as occupancy covariates but were not supported in the top‐ranked models based on AIC and are therefore not discussed further. The top‐ranked model, p (Season + Effort) ψ (Distance to Water Body + Slope + Distance to Hunting Camps). Occupancy decreased with increasing distance from water bodies (*β* = −0.82, SE = 0.33, *p* = 0.014; Figure 2a). In contrast, slope had little apparent effect on occupancy (*β* = −0.05, SE = 0.28, *p* = 0.83; Figure 2b), showing only a slight positive trend. Occupancy increased with distance from hunting camps (*β* = 0.65, SE = 0.31, *p* = 0.036; Figure 2c), suggesting reduced habitat use near areas with hunting activity. Overall, these results indicate that environmental and anthropogenic covariates primarily drive occupancy patterns at the park scale.

### 3.3 Seasonal Variation in Habitat Use

**FIGURE 3:**
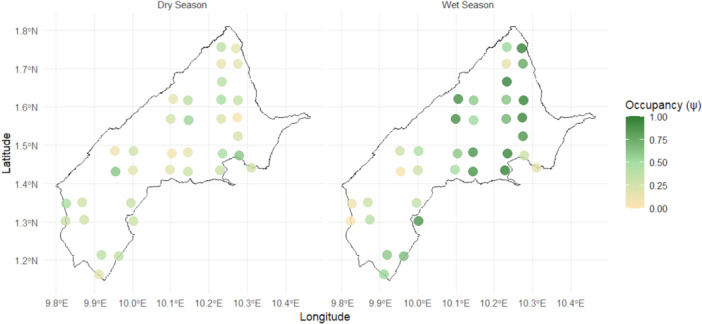
Spatial distribution of mandrill occupancy probability (ψ) across camera‐trap stations in Monte Alén National Park during the dry season (left) and wet season (right). Color indicates site‐specific occupancy probability, with darker green representing higher values. Coordinates of occupancy locations are shown approximately to avoid disclosing precise positions for conservation purposes.

To further explore seasonal variation at the site level, site‐level analyses revealed distinct seasonal patterns in habitat use. Paired comparisons of occupancy probabilities at the same camera sites showed higher occupancy during the wet season (mean difference = 0.30; paired *t*‐test: *t*₃₄ = 4.77, *p* < 0.001). The variance in occupancy among camera sites was greater in the wet season (0.086) than in the dry season (0.027), indicating that mandrills concentrated their activity in specific high‐use sites during the wet season, whereas distribution was more even across sites in the dry season (Figure 3). These results demonstrate clear seasonal changes in site‐level habitat use, while overall park‐wide occupancy remained relatively stable.

## 4 Discussion

Understanding how large‐bodied primates use space in heterogeneous and human‐impacted landscapes is central to primate ecology and conservation, as spatial and temporal patterns of habitat use reflect trade‐offs between access to resources and avoidance of risks, such as predation and hunting (Bryson‐Morrison et al. 2017; Hamilton 2019; Mayor et al. 2009). In tropical forests, where seasonality and human activities strongly shape resource availability, these patterns provide key insights into species persistence under increasing anthropogenic pressure. Within this context, our study examined how environmental and human factors influence mandrill habitat use across MANP. By linking occupancy patterns to ecological and socio‐economic drivers, we highlight this vulnerable species responds to hunting and landscape heterogeneity, contributing to a broader understanding of primate space use in disturbed tropical ecosystems.

### 4.1 Site Use and Seasonal Patterns

Mandrills appear to use the landscape unevenly, with activity concentrated in certain areas depending on local conditions. Site‐level analysis showed higher variance in occupancy during the wet season, whereas occupancy was more evenly distributed across sites during the dry season. This pattern may indicate seasonal shifts in space use, possibly reflecting changes in the spatial distribution of resources, although resource availability was not directly measured in this study. In tropical forests, seasonal variation in fruit availability influences primate space use, and the flexible, omnivorous diet of mandrills may facilitate seasonal adjustments in habitat use (Hongo et al. 2018).

Although detailed data on vegetation composition and fruiting in MANP are limited, certain tree species known to fruit primarily in the rain season in Equatorial Guinea, such as *Dacryodes edulis*, *Irvingia gabonensis*, and *Aucoumea klaineana*, may create temporal resource hotspots (Orwa et al. 2009). Similar patterns have been observed in other Central African forests, where mandrills adjust their movement and foraging behavior in response to seasonal resource changes. For example, in Moukalaba‐Doudou National Park (Gabon), mandrills exhibited longer day ranges and concentrated movement patterns during fruiting seasons, likely tracking clumped fruit resources, while showing shorter and more evenly distributed movements during non‐fruiting periods (Hongo et al. 2021). Similarly, in Lopé National Park (Gabon), wet‐season fruit availability led mandrills to focus foraging on fruit, whereas in the dry season, they expanded their diet to fallback foods and traveled further to locate dispersed resources (Bauld et al. 2025). These findings highlight the importance of considering both mean occupancy and spatial variance when assessing habitat use and seasonal ecology. Future research incorporating vegetation surveys, fruiting phenology, and direct foraging observations would provide further insight into the ecological drivers of these site‐specific seasonal patterns.

### 4.2 Environmental Influences: Water Bodies and Slope

The water covariate in this study included only main rivers, excluding smaller streams because of spatial data limitations. Mandrills are likely to use smaller, unmapped water sources, which may have led to an underestimation of suitable habitats or occupancy. Access to water is a key factor shaping mandrill habitat selection, supporting hydration, thermoregulation, and access to riparian food resources (Thompson and Hermann 2024). Lahm (1986) observed that mandrills frequently occurred on slopes and plateaus above streams or rivers, with up to 60% of identified food sources obtained from riparian habitats. Although terrestrial, mandrills occasionally exploit aquatic resources, having been observed fishing in small streams (Jouventin 1975), and remains of fish, crabs, and freshwater shrimp near mandrill tracks suggest opportunistic foraging. Similar behavior has been reported in closely related Bioko Island drills (*Mandrillus leucophaeus*), where crustaceans composed up to 61% of animal remains in fecal samples (Owens et al. 2015). Together, these findings highlight the ecological importance of water availability in shaping mandrill habitat use and spatial activity patterns.

Slope appears to have a more limited influence on mandrill distribution. Their generalist diet, wide‐ranging foraging strategies, and physical adaptations allow them to navigate diverse terrains effectively (Lahm 1986). As semi‐terrestrial foragers, mandrills can move across uneven forest floors, and moderate inclines appear to pose minimal limitation on movement or habitat use (Emmons et al. 1983). Large home ranges and long‐distance travel capacity may buffer against localized topographic variation (Hoshino et al. 1984), while robust bodies and elongated limbs, support efficient movement through complex landscapes, enabling access to dispersed resources (Lahm 1986).

### 4.3 Human Pressures: Hunting and Avoidance

Mandrill occupancy is strongly influenced by proximity to hunting camps, reflecting both avoidance behavior and direct impacts. Mandrills tend to occupy areas farther from known hunting sites because of a combination of active avoidance and reduced local densities. In MANP, hunting is legally allowed in some park areas, and weak enforcement likely contributes to these patterns. The absence of physical boundaries between the park and surrounding land allows hunters and wildlife to move freely, complicating management (Kümpel et al. 2008; Vizzuality 2024).

Hunting can directly reduce mandrill group sizes, lowering detectability through fewer visual encounters and vocalizations (Bessone et al. 2023). More broadly, mandrills show patchy distributions and variable densities, likely reflecting spatial variation in hunting pressure, consistent with the *Cercocebus* and *Mandrillus* Conservation Action Plan 2024–2028 (Dempsey et al. 2024). Economic incentives strongly drive hunting, as market surveys conducted in Sendje indicate that mandrill meat commands a relatively high price compared with other primates (850 CFA/kg vs. 500 CFA/kg for black colobus (*Colobus satanas*) and 300 CFA/kg for chimpanzees), reinforcing their attractiveness to hunters (Kümpel et al. 2008). Juveniles are often targeted, adult males are somewhat overrepresented, and larger‐bodied individuals are more likely to be caught in snares (Lindsey et al. 2011, 2013). Mandrills are among the most frequently targeted primates, with 90% probability of being pursued when encountered (Kümpel 2006; Kümpel et al. 2008; Rist et al. 2009).

Other Central African primates show adaptive behaviors to reduce hunting risk. Species such as gray‐cheeked mangabey (*Lophocebus albigena*), red‐capped mangabey (*Cercocebus torquatus*), mustached monkey (*Cercopithecus cephus*), and putty‐nosed monkey (*Cercopithecus nictitans*) delay alarm calls or become more secretive in heavily hunted areas (Croes et al. 2007). These patterns suggest that mandrills are likely to exhibit similar avoidance strategies, consistent with their higher occupancy probabilities at greater distance from hunting camps.

### 4.4 Socio‐Economic Drivers and Conservation Implications

Socio‐economic factors strongly influence mandrill occupancy near hunting camps, highlighting the link between local livelihoods and wildlife distribution (Section 4.3). Equatorial Guinea's reliance on oil and gas, which once accounted for over 95% of government revenue, has declined from 328,690 barrels/day in 2004 to ~54,881 barrels/day in 2023 (Gusarov 2020; CEIC Data n.d.), reducing employment and driving rural households toward wild meat for protein and income. Mandrill meat is particularly valuable, sustaining hunting camps despite legal restrictions within 5 km of the park (Law No. 8/1988), largely because enforcement is minimal (Avoro and Eneme Efua 2002; Kümpel 2006).

While mandrill occupancy declines near hunting camps because of hunting pressure and avoidance behavior, removing or relocating camps without providing alternative livelihoods risks exacerbating poverty and food insecurity, potentially undermining conservation compliance (Meyer and Börner 2022; Salerno et al. 2020; Dickson 2003; Egbe 2001).

A range of alternative livelihoods could reduce dependence on wild meat, though feasibility depends on cultural acceptance, economic viability, and ecological sustainability. Small‐scale livestock, particularly poultry, is widely reared in Equatorial Guinea and provides both protein and income (Allebone‐Webb 2009; Keylock 2002; CTA Technical Centre for Agriculture and Rural Cooperation 2004). Goats and trypanosomiasis‐resistant dwarf cattle are feasible where veterinary support exists (Kümpel 2006; Fa 2000). Fish and aquaculture, including integrated fish‐livestock systems, offer locally produced protein and income but require technical support and investment (Evans et al. 2011; Machena and Moehl 2001). Most fish consumed is imported (Food and Agriculture Organization of the United Nations 2003; Kümpel 2006), creating both a protein gap and an opportunity for sustainable development. Lessons from Rwanda and Malawi suggest that combining fishponds with poultry, rabbits, or goats, can provide multiple protein sources while encouraging active community participation (Evans et al. 2011).

Other low‐impact, environmentally sustainable activities, such as farming fast‐growing wild species (e.g., snails), beekeeping, or butterfly farming, can supplement income with minimal inputs (Evans et al. 2011; Ndah et al. 2017). Community‐based ecotourism also has potential for employment and wildlife conservation, though infrastructure and equitable benefit‐sharing remain challenges (Forje et al. 2020, 2021; Snyman et al. 2023).

Integrating small‐scale livestock, aquaculture, wildlife farming, low‐impact income activities, and ecotourism offers a feasible pathway to reduce hunting pressure while sustaining rural livelihoods. Conservation strategies that balance ecological priorities with socio‐economic realities are essential for ensuring long‐term survival of mandrills in MANP.

## 5 Concluding Remarks

This study provides key insights into the factors shaping mandrill occupancy within MANP, highlighting the importance of proximity to water bodies and the clear impact of hunting pressure. While park‐wide occupancy showed no strong seasonal differences, site‐level analyses revealed that mandrills concentrate activity in resource‐rich areas during the wet season, demonstrating flexible, site‐specific habitat use.

Proximity to hunting camps was as a significant predictor of occupancy, with lower detection near camps reflecting both direct hunting and behavioral avoidance. These patterns emphasize the role of socio‐economic drivers: hunting persists because mandrill meat is valuable, enforcement is limited, and alternative livelihoods are scarce.

Effective conservation must balance ecological priorities with community needs. In the short term, stronger regulation of sensitive park areas, particularly around hunting camps and critical water sources, is essential. In the long term, promoting alternatives, such as small‐scale livestock, aquaculture, wildlife farming, low‐impact income‐generating activities, and community‐based ecotourism, can reduce reliance on wild meat while supporting rural livelihoods. Addressing both immediate threats and underlying socio‐economic factors is crucial for developing sustainable strategies to ensure the long‐term survival of mandrills in MANP.

## Author Contributions

**Tania Guzmán‑Santillán:** investigation, methodology, data curation, formal analysis, writing – original draft, writing – review & editing. **Salvador Mandujano:** methodology, formal analysis, writing – review & editing. **Edward Wright:** conceptualization, methodology, writing – review & editing. **David Fernández:** conceptualization, methodology. **Juan‑Cruz Ondo Nze Avomo:** conceptualization, methodology. **Fidel Esono Mba Eyono:** conceptualization. **Timothy Bray:** conceptualization, supervision, funding acquisition, project administration, validation, writing – review & editing.

## Supporting information

Appendix 1 Supporting Information R2.

Research Data.
