# Supplementary material for: Impact of Environmental and Anthropogenic Factors on Mandrill (Mandrillus sphinx) Occupancy and Habitat Use in Monte Alén National Park, Equatorial Guinea
Source: Am J Primatol. 2026 Feb 17;88(2):e70125. doi: 10.1002/ajp.70125 (PMC12910328; doi:10.1002/ajp.70125)
Supplement: Supplementary file 1 — Appendix 1 Supporting Information R2. [file AJP-88-e70125-s002.docx]

**R script: Single-species, single-season occupancy modeling**

options(repos = c(CRAN = "https://cloud.r-project.org"))

install.packages("knitr")

install.packages("rmarkdown")

install.packages("evaluate")

install.packages("MuMIn")

install.packages("unmarked")

install.packages("AICcmodavg")

install.packages("purrr")

library(tidyverse)

library(unmarked)

library(MuMIn)

library(readxl)

library(AICcmodavg)

library(dplyr)

library(lubridate)

library(purrr)

options(na.action = "na.fail")

# Detection history data collapsed into nine-days intervals

DH <- read_excel("DH_r 1 año.xlsx")

det_hist <- DH %>%

column_to_rownames("Stations")%>%

mutate(across(c(day01_day09, day10_day18, day19_day27, day28_day36, day37_day45),

as.numeric))

head(det_hist)

tail(det_hist)

# Calculate naive occupancy estimate

naive_occupancy <- sum(apply(det_hist, 1, function(x) any(x == 1, na.rm = TRUE))) / nrow(det_hist)

naive_occupancy_percent <- naive_occupancy * 100

cat("Naive Occupancy:", round(naive_occupancy_percent, 2), "%\n")

# Import site covariates

Site_covs<- read_excel("Site_covs2.xlsx") %>%

dplyr::select(-Sum,-Det,-utm_x,-utm_y)

colnames(Site_covs) <- trimws(colnames(Site_covs))

print(colnames(Site_covs))

head(Site_covs)

tail(Site_covs)

# Import sampling covariates

Covs <- read_excel("Covs2.xlsx")

head(Covs)

tail(Covs)

# Scale numeric covariates

scale_vec <- function(x) {

vec <- as.vector(scale(x))

return(vec)

}

cov.num <- Covs %>%

dplyr::select(where(is.numeric)) %>%

dplyr::select(-X, -Y) %>%

mutate(across(everything(), scale_vec))

head(cov.num)

tail(cov.num)

# Separate factor and spatial covariates

cov.fac <- Covs %>% dplyr::select(where(is.character))

cov.xy <- Covs %>% dplyr::select(X, Y)

# Combine all covariates into one dataset

covars <- data.frame(cov.xy, cov.fac, cov.num)

head(covars)

tail(covars)

# Convert selected columns to factors

covars$Season <- factor(covars$Season)

covars$Station <- factor(covars$Station)

covars$Stations <- factor(covars$Stations)

covars$Model <- factor(covars$Model)

covars$Season_Year <- factor(covars$Season_Year)

str(covars)

# Plot mandrill detections based on camera-trap data

DH1 <- read_excel("DHPlot.xlsx")

det_hist1 <- DH1 %>%

mutate(across(c(day01_day09, day10_day18, day19_day27, day28_day36, day37_day45),

as.numeric))

det_hist1 <- det_hist1 %>%

mutate(total_detections = rowSums(select(.,

c(day01_day09, day10_day18,

day19_day27, day28_day36, day37_day45)),

na.rm = TRUE))

det_summary1 <- det_hist1 %>%

group_by(Station) %>%

summarise(total_detections = sum(total_detections, na.rm = TRUE))

print(det_summary1)

print (det_summary1, n=36)

coords1 <- covars %>%

select(Station, X, Y) %>%

distinct(Station, .keep_all = TRUE)

det_map1 <- det_summary1 %>%

left_join(coords1, by = "Station")

library(sf)

boundary <- st_read("MAPA 2.shp")

det_map1 <- det_map1 %>% filter(!is.na(X) & !is.na(Y))

det_map1 <- det_map1 %>% filter(!is.na(X) & !is.na(Y))

det_map1_sf <- st_as_sf(det_map1, coords = c("X", "Y"), crs = 32632)

det_map1_wgs84 <- st_transform(det_map1_sf, crs = 4326)

ggplot() +

geom_sf(data = boundary, fill = NA, color = "black", size = 1) +

geom_sf(data = det_map1_wgs84, aes(size = total_detections, color = total_detections), alpha = 0.8) +

scale_size_continuous(range = c(2, 6)) +

scale_color_gradientn(

colors = c("#FFE5B4", "#A6DDA3", "#2E7D32"))+

theme_minimal() +

labs(x = "Longitude",

y = "Latitude",

size = "No. of detections",

color = "No. of detections") +

coord_sf() +

theme(legend.position = "right",

plot.margin = margin(t = 50, r = 50, b = 20, l = 20),

legend.title = element_text(size = 10),

legend.text = element_text(size = 8),

axis.text.x = element_text(angle = 45, hjust = 1),

axis.text.y = element_text(size = 8))

# Import effort data

Effort <- read_excel("Effort2.xlsx")%>%

column_to_rownames("Stations")%>%

mutate(across(c(day01_day09, day10_day18, day19_day27, day28_day36, day37_day45),

as.numeric))

head(Effort)

tail(Effort)

# Create unmarked data frame for occupancy analysis

vuf <- unmarkedFrameOccu(y= det_hist,

siteCovs = covars,

obsCovs = list(Effort= Effort))

summary(vuf)

plot(vuf)

# Calculate total effective camera-trap days

df <- read_excel("Data frame_.xlsx")

df <- df %>%

mutate(

Date_placement = as.Date(Date_placement),

Date_removal = as.Date(Date_removal),

Problem1_from = as.Date(Problem1_from),

Problem1_to = as.Date(Problem1_to),

Problem2_from = as.Date(Problem2_from),

Problem2_to = as.Date(Problem2_to),

Problem3_from = as.Date(Problem3_from),

Problem3_to = as.Date(Problem3_to)

)

calc_active_days <- function(start, end, probs) {

full_period <- seq.Date(start, end - 1, by = "day")

for (i in seq(1, length(probs), by = 2)) {

from <- probs[[i]]

to <- probs[[i + 1]]

if (!is.na(from) && !is.na(to)) {

problem_range <- seq.Date(from, to - 1, by = "day")

full_period <- setdiff(full_period, problem_range)

}

}

length(full_period)

}

df$active_days <- pmap_int(df, function(Station, Date_placement, Date_removal,

Problem1_from, Problem1_to,

Problem2_from, Problem2_to,

Problem3_from, Problem3_to) {

probs <- list(Problem1_from, Problem1_to,

Problem2_from, Problem2_to,

Problem3_from, Problem3_to)

calc_active_days(Date_placement, Date_removal, probs)

})

total_days <- sum(df$active_days)

cat("Total effective camera-trap days:", total_days, "\n")

# Summarize active days per station

df %>% select(Station, active_days)

print (df, n=36)

print(df %>% select(Station, active_days), n = Inf)

mean(df$active_days)

sd(df$active_days)

library(ggplot2)

ggplot(df, aes(x = reorder(Station, active_days), y = active_days)) +

geom_col(fill = "#3CB371", width = 0.7) +

coord_flip() +

labs(title = "Camera Trap Activity by Station",

x = "Camera Trap Station",

y = "Number of Active Days") +

geom_hline(yintercept = mean(df$active_days), linetype = "dashed", color = "grey30", size = 0.8) +

theme_minimal(base_size = 13) +

theme(

plot.title = element_text(face = "bold", hjust = 0.5),

axis.text = element_text(color = "black"),

panel.grid.major.y = element_blank(),

panel.grid.minor = element_blank()

)

# Calculate total number of independent detection

total_detections <- sum(vuf@y, na.rm = TRUE)

cat("Total detections:", total_detections, "\n")

detections_df <- det_summary1 %>% rename(detections = total_detections)

effort_df <- df %>% select(Station, active_days)

summary_df <- left_join(detections_df, effort_df, by = "Station")

# Calculate RAI (events per 100 camera-trap days)

summary_df <- summary_df %>%

mutate(RAI_per_100_days = (detections / active_days) * 100)

# Descriptive statistics

total_days <- sum(summary_df$active_days, na.rm = TRUE)

total_detections <- sum(summary_df$detections, na.rm = TRUE)

mean_RAI <- mean(summary_df$RAI_per_100_days, na.rm = TRUE)

median_RAI <- median(summary_df$RAI_per_100_days, na.rm = TRUE)

min_RAI <- min(summary_df$RAI_per_100_days, na.rm = TRUE)

max_RAI <- max(summary_df$RAI_per_100_days, na.rm = TRUE)

sd_RAI <- sd(summary_df$RAI_per_100_days, na.rm = TRUE)

print(summary_df)

cat("Total effective camera-trap days:", total_days, "\n")

cat("Total detections:", total_detections, "\n")

cat("Mean RAI:", mean_RAI, "\n")

cat("Median RAI:", median_RAI, "\n")

cat("Min RAI:", min_RAI, "\n")

cat("Max RAI:", max_RAI, "\n")

cat("SD RAI:", sd_RAI, "\n")

# Mandrill detection map

# Merge RAI into map dataframe

det_map1 <- det_map1 %>%

left_join(summary_df %>% select(Station, RAI_per_100_days), by = "Station")

# Convert to sf object and transform CRS

det_map1_sf <- st_as_sf(det_map1, coords = c("X", "Y"), crs = 32632)

det_map1_wgs84 <- st_transform(det_map1_sf, crs = 4326)

ggplot() +

geom_sf(data = boundary, fill = NA, color = "black", size = 1) +

geom_sf(data = det_map1_wgs84,

aes(size = RAI_per_100_days, color = RAI_per_100_days), alpha = 0.8) +

scale_size_continuous(range = c(2, 6)) +

scale_color_gradientn(colors = c("#FFE5B4", "#A6DDA3", "#2E7D32")) +

theme_minimal() +

labs(x = "Longitude",

y = "Latitude",

size = "RAI (events/100 days)",

color = "RAI (events/100 days)") +

coord_sf() +

theme(legend.position = "right",

plot.margin = margin(t = 50, r = 50, b = 20, l = 20),

legend.title = element_text(size = 10),

legend.text = element_text(size = 8),

axis.text.x = element_text(angle = 45, hjust = 1),

axis.text.y = element_text(size = 8))

# Fit detection probability models

m0 <- occu(~1 ~1, data = vuf)

md1 <- occu(~Effort ~1, data = vuf)

md2 <- occu(~Model ~1, data = vuf)

md3 <- occu(~Season ~1, data = vuf)

md4 <- occu(~Season * Model ~1, data = vuf)

md5 <- occu(~Season * Effort ~1, data = vuf)

md6 <- occu(~ Season + Model ~1, data = vuf)

md7 <- occu(~ Season + Effort ~1, data = vuf)

det_cadn <- list(m0,md1,md2,md3, md4, md5, md6, md7)

det_mnames <- c("p(.) psi(.)"

,"p(Effort) psi(.)"

,"p(Model) psi(.)"

,"p(Season) psi(.)"

,"p(Season * Model) psi(.)"

,"p(Season* Effort) psi(.)"

, "p(Season + Model) psi(.)"

, "p(Season + Effort) psi(.)")

(det_AIC <- aictab(cand.set = det_cadn,

modnames = det_mnames,

second.ord = F,

sort = T))

# Select the best detection model

summary(md7)

# Goodness-of-fit test

set.seed(123)

pdf(NULL)

m0_gof <- mb.gof.test(md7)

dev.off()

summary(m0_gof)

coef(md7)

backTransform(linearComb(md7, coefficients = c(1), type = "state"))

backTransform(linearComb(md7, coefficients = c(0,1,1), type = "det"))

# Plot predicted detection probability as a function of effort

effort_values <- seq(min(Effort, na.rm = TRUE), max(Effort, na.rm = TRUE), length.out = 100)

pred_effort <- predict(md1, type = "det", newdata = data.frame(Effort = effort_values))

pred_effort_df <- data.frame(

Effort = effort_values,

Pred = pred_effort$Predicted,

SE = pred_effort$SE

)

pred_effort_df$Lower <- pred_effort_df$Pred - 1.96 * pred_effort_df$SE

pred_effort_df$Upper <- pred_effort_df$Pred + 1.96 * pred_effort_df$SE

min(Effort, na.rm = TRUE)

max(Effort, na.rm = TRUE)

ggplot(pred_effort_df, aes(x = Effort, y = Pred)) +

geom_line(color = "grey") +

geom_ribbon(aes(ymin = Lower, ymax = Upper), alpha = 0.2, fill = "steelblue") +

labs(x = "Effort", y = "Detection probability") +

theme_minimal()

# Plot predicted detection probability by season

season_levels <- unique(covars$Season)

season_pred_data <- data.frame(Season = season_levels)

pred_season <- predict(md3, type = "det", newdata = season_pred_data)

pred_season_df <- data.frame(

Season = season_levels,

Pred = pred_season$Predicted,

SE = pred_season$SE

)

pred_season_df$Lower <- pred_season_df$Pred - 1.96 * pred_season_df$SE

pred_season_df$Upper <- pred_season_df$Pred + 1.96 * pred_season_df$SE

ggplot(pred_season_df, aes(x = Season, y = Pred)) +

geom_point() +

geom_errorbar(aes(ymin = Lower, ymax = Upper), width = 0.2) +

labs(x = "Season", y = "Detection probability") +

theme_minimal()

# Fit occupancy models with site covariates

moc1 <- occu( ~ Season + Effort ~ Dis_PB, data = vuf)

moc2 <- occu(~ Season + Effort ~ Dis_HS, data = vuf)

moc3 <- occu(~ Season + Effort ~ Dis_Vil, data = vuf)

moc4 <- occu(~ Season + Effort ~ Dis_WB, data = vuf)

moc5 <- occu(~ Season + Effort ~ Slope, data = vuf)

moc6 <- occu(~ Season + Effort ~ Dis_PB + Slope, data = vuf)

moc7 <- occu(~ Season + Effort ~ Dis_HS + Slope, data = vuf)

moc8 <- occu(~ Season + Effort ~ Dis_Vil + Slope, data = vuf)

moc9 <- occu(~ Season + Effort ~ Dis_WB + Slope, data = vuf)

moc10 <- occu(~ Season + Effort ~ Dis_WB + Slope + Dis_HS, data = vuf)

oc_cadn <- list(m0,moc1,moc2,moc3,moc4,moc5, moc6, moc7, moc8, moc9, moc10)

oc_mnames <- c("p(.) psi(.)"

,"p(Season + Effort) psi(Dis_PB)"

,"p(Season + Effort) psi(Dis_HS)"

,"p(Season + Effort) psi(Dis_Vil)"

,"p(Season + Effort) psi(Dis_WB)"

,"p(Season + Effort) psi(Slope)"

,"p(Season + Effort) psi(Dis_PB + Slope)"

,"p(Season + Effort) psi(Dis_HS + Slope)"

,"p(Season + Effort) psi(Dis_Vil + Slope)"

,"p(Season + Effort) psi(Dis_WB + Slope)"

,"p(Season + Effort) psi(Dis_WB + Slope + Dis_HS)"

)

(oc_AIC_covs <- aictab(cand.set = oc_cadn,

modnames = oc_mnames,

second.ord = F,

sort = T))

# Select the best occupancy model

summary(moc10)

# Goodness-of-fit test for best occupancy model

set.seed(123)

pdf(NULL)

m9_gof <- mb.gof.test(moc10)

dev.off()

summary(m9_gof)

backTransform(linearComb(moc10, coefficients = c(1, 1, 1, 1), type = "state"))

backTransform(linearComb(moc10, coefficients = c(1, 1, 1), type = "det"))

# Extract confidence intervals and coefficients

bestmods_ic <- list(moc10) %>%

lapply(.,confint, type= "state") %>%

lapply(as.data.frame) %>%

reduce(bind_rows) %>%

rownames_to_column(var= "Parameter")

bestmods_coef <- list(moc10) %>%

lapply(., coef, type = "state") %>%

lapply(as.data.frame) %>%

reduce(bind_rows) %>%

rownames_to_column(var = "Parameter") %>%

left_join(bestmods_ic) %>%

mutate(model = "moc10") %>% # Assign a single value instead of a vector

mutate(parameter2 = case_when(

str_detect(Parameter, "(Int)...") ~ "Psi",

TRUE ~ "no")) %>%

filter(parameter2 != "Psi") %>%

rename(coef = "X[[i]]",

low = "0.025",

high ="0.975")

# Plot model coefficients

(models <- ggplot(bestmods_coef, aes(x = coef, y = Parameter, xmin = low, xmax = high)) +

geom_pointrange(aes(col = model), size = 1.2) +

geom_vline(xintercept = 0, linetype = "dashed", color = "black") +

theme_bw() +

scale_color_manual(values = c("red")) +

theme(text = element_text(size = 14)))

summary(moc10)

coef(moc10, type = "state")

coef(moc10, type = "det")

# Occupancy prediction for psi(Dis_WB) with p(Season + Effort)

# Extract original covariate values

dis_wb_orig <- Covs$Dis_WB

# Calculate mean and SD from the raw data

mean_dis_wb <- mean(dis_wb_orig, na.rm = TRUE)

sd_dis_wb <- sd(dis_wb_orig, na.rm = TRUE)

# Generate a sequence on the original scale

dis_wb_seq <- seq(min(dis_wb_orig, na.rm = TRUE), max(dis_wb_orig, na.rm = TRUE), length.out = 100)

# Standardize the sequence for prediction

dis_wb_scaled <- (dis_wb_seq - mean_dis_wb) / sd_dis_wb

# Use mean (zero-scaled) values for the other covariates

slope_scaled_mean <- 0

dis_hs_scaled_mean <- 0

# Create new data for prediction

newdata_dis_wb <- data.frame(

Dis_HS = dis_hs_scaled_mean,

Dis_WB = dis_wb_scaled,

Slope = slope_scaled_mean

)

# Predict occupancy probability

pred_dis_wb <- predict(moc10, type = "state", newdata = newdata_dis_wb)

# Build dataframe for plotting

pred_df_dis_wb <- data.frame(

Dis_WB = dis_wb_seq,

Pred = pred_dis_wb$Predicted,

Lower = pred_dis_wb$Predicted - 1.96 * pred_dis_wb$SE,

Upper = pred_dis_wb$Predicted + 1.96 * pred_dis_wb$SE

)

# Plot predicted occupancy

plot(pred_df_dis_wb$Pred ~ pred_df_dis_wb$Dis_WB, type = "l", lwd = 3, col = "forestgreen",

ylim = c(0, 1), las = 1, ylab = "Occupancy Probability (Ψ)",

xlab = "Distance to water bodies (km)", frame = FALSE, xaxt = "n")

axis(1, at = pretty(pred_df_dis_wb$Dis_WB, n = 5))

matlines(pred_df_dis_wb$Dis_WB, cbind(pred_df_dis_wb$Lower, pred_df_dis_wb$Upper),

lty = 1, lwd = 1, col = "grey")

# Occupancy prediction for psi(Dis_HS) with p(Season + Effort)

# Extract original covariate values

dis_hs_orig <- Covs$Dis_HS

# Calculate mean and SD from the raw data

mean_dis_hs <- mean(dis_hs_orig, na.rm = TRUE)

sd_dis_hs <- sd(dis_hs_orig, na.rm = TRUE)

# Generate a sequence on the original scale

dis_hs_seq <- seq(min(dis_hs_orig, na.rm = TRUE), max(dis_hs_orig, na.rm = TRUE), length.out = 100)

# Standardize the sequence for prediction

dis_hs_scaled <- (dis_hs_seq - mean_dis_hs) / sd_dis_hs

# Use mean (zero-scaled) values for the other covariates

slope_scaled_mean <- 0

dis_wb_scaled_mean <- 0

# Create new data for prediction

newdata_dis_hs <- data.frame(

Dis_HS = dis_hs_scaled,

Dis_WB = dis_wb_scaled_mean,

Slope = slope_scaled_mean

)

# Predict occupancy probability

pred_dis_hs <- predict(moc10, type = "state", newdata = newdata_dis_hs)

# Build dataframe for plotting

pred_df_dis_hs <- data.frame(

Dis_HS = dis_hs_seq,

Pred = pred_dis_hs$Predicted,

Lower = pred_dis_hs$Predicted - 1.96 * pred_dis_hs$SE,

Upper = pred_dis_hs$Predicted + 1.96 * pred_dis_hs$SE

)

# Plot predicted occupancy

plot(pred_df_dis_hs$Pred ~ pred_df_dis_hs$Dis_HS, type = "l", lwd = 3, col = "forestgreen",

ylim = c(0, 1), las = 1, ylab = "Occupancy Probability (Ψ)",

xlab = "Distance to hunting camps (km)", frame = FALSE, xaxt = "n")

axis(1, at = pretty(pred_df_dis_hs$Dis_HS, n = 5))

matlines(pred_df_dis_hs$Dis_HS, cbind(pred_df_dis_hs$Lower, pred_df_dis_hs$Upper),

lty = 1, lwd = 1, col = "grey")

#Occupancy prediction for psi(Slope) with p(Season + Effort)

# Extract original covariate values

slope_orig <- Covs$Slope

# Calculate mean and SD from the raw data

mean_slope <- mean(slope_orig, na.rm = TRUE)

sd_slope <- sd(slope_orig, na.rm = TRUE)

# Generate a sequence on the original scale

slope_seq <- seq(min(slope_orig, na.rm = TRUE), max(slope_orig, na.rm = TRUE), length.out = 100)

# Standardize the sequence for prediction

slope_scaled <- (slope_seq - mean_slope) / sd_slope

# Use mean (zero-scaled) values for the other covariates

dis_hs_scaled_mean <- 0

dis_wb_scaled_mean <- 0

# Create new data for prediction

newdata_slope <- data.frame(

Dis_HS = dis_hs_scaled_mean,

Dis_WB = dis_wb_scaled_mean,

Slope = slope_scaled

)

# Predict occupancy probability

pred_slope <- predict(moc10, type = "state", newdata = newdata_slope)

# Build dataframe for plotting

pred_df_slope <- data.frame(

Slope = slope_seq,

Pred = pred_slope$Predicted,

Lower = pred_slope$Predicted - 1.96 * pred_slope$SE,

Upper = pred_slope$Predicted + 1.96 * pred_slope$SE

)

# Plot predicted occupancy

plot(pred_df_slope$Pred ~ pred_df_slope$Slope, type = "l", lwd = 3, col = "forestgreen",

ylim = c(0, 1), las = 1, ylab = "Occupancy Probability (Ψ)",

xlab = "Slope (%)", frame = FALSE, xaxt = "n")

axis(1, at = pretty(pred_df_slope$Slope, n = 5))

matlines(pred_df_slope$Slope, cbind(pred_df_slope$Lower, pred_df_slope$Upper),

lty = 1, lwd = 1, col = "grey")

# Site-level occupancy per season and paired test

# Extract site- and observation-level covariates

sc <- siteCovs(vuf)

obs <- obsCovs(vuf)

# Identify sites by season

wet_idx <- which(sc$Season == "Rain season")

dry_idx <- which(sc$Season == "Dry season")

# Subset detection histories by season

y <- getY(vuf)

y_wet <- y[wet_idx, ]

y_dry <- y[dry_idx, ]

# Subset site covariates

sc_wet <- sc[wet_idx, ]

sc_dry <- sc[dry_idx, ]

# Subset observation covariates

obs_wet <- obs[wet_idx, , drop = FALSE]

obs_dry <- obs[dry_idx, , drop = FALSE]

# Determine the number of survey occasions

n_occasions <- ncol(y_wet)

# Expand survey effort across all occasions

obs_wet_mat <- matrix(rep(obs_wet$Effort, each = n_occasions), nrow = nrow(y_wet), ncol = n_occasions)

obs_dry_mat <- matrix(rep(obs_dry$Effort, each = n_occasions), nrow = nrow(y_dry), ncol = n_occasions)

# Convert to list

obs_wet_list <- list(Effort = obs_wet_mat)

obs_dry_list <- list(Effort = obs_dry_mat)

# Create unmarked frames for each season

umf_wet <- unmarkedFrameOccu(y = y_wet, siteCovs = sc_wet, obsCovs = obs_wet_list)

umf_dry <- unmarkedFrameOccu(y = y_dry, siteCovs = sc_dry, obsCovs = obs_dry_list)

# Fit occupancy models separately for wet and dry seasons

fm_wet <- occu(~ Effort ~ Dis_WB + Slope + Dis_HS, data = umf_wet)

fm_dry <- occu(~ Effort ~ Dis_WB + Slope + Dis_HS, data = umf_dry)

# Estimate site-level occupancy probabilities

psi_wet <- predict(fm_wet, type = "state")$Predicted

psi_dry <- predict(fm_dry, type = "state")$Predicted

# Extract station names for each season

stations_wet <- sc_wet$Station

stations_dry <- sc_dry$Station

# Identify stations present in both seasons

common_stations <- intersect(sc_wet$Station, sc_dry$Station)

# Align occupancy probabilities by common stations

psi_wet_common <- psi_wet[match(common_stations, sc_wet$Station)]

psi_dry_common <- psi_dry[match(common_stations, sc_dry$Station)]

# Paired t-test comparing wet vs. dry season occupancy

t_test_result <- t.test(psi_wet_common, psi_dry_common, paired = TRUE)

print(t_test_result)

# Compare variance and coefficient of variation (CV)

var_wet <- var(psi_wet)

var_dry <- var(psi_dry)

cv_wet <- sd(psi_wet)/mean(psi_wet)

cv_dry <- sd(psi_dry)/mean(psi_dry)

cat("Variance wet:", var_wet, "dry:", var_dry, "\n")

cat("CV wet:", cv_wet, "dry:", cv_dry, "\n")

# Spatial visualization of site-level occupancy

# Clean and standardize station IDs

sc$Station <- trimws(as.character(sc$Station))

sc_wet$Station <- trimws(as.character(sc_wet$Station))

sc_dry$Station <- trimws(as.character(sc_dry$Station))

# Identify stations sampled in both seasons

common_stations <- intersect(sc_wet$Station, sc_dry$Station)

length(common_stations) # should be 35

# Subset coordinates for common stations

site_coords_common <- sc[sc$Station %in% common_stations, ]

dim(site_coords_common)

# Reorder coordinates and occupancy estimates by station order

site_coords_common <- site_coords_common[match(common_stations, site_coords_common$Station), ]

psi_wet_common <- psi_wet[match(common_stations, sc_wet$Station)]

psi_dry_common <- psi_dry[match(common_stations, sc_dry$Station)]

# Combine into a single data frame

occ_map <- data.frame(

Station = site_coords_common$Station,

X = site_coords_common$X,

Y = site_coords_common$Y,

psi_wet = psi_wet_common,

psi_dry = psi_dry_common

)

# Convert to sf object

occ_map_sf <- st_as_sf(occ_map, coords = c("X", "Y"), crs = 32632)

occ_map_wgs84 <- st_transform(occ_map_sf, crs = 4326)

# Reshape to Long format for plotting

occ_map_long <- occ_map_wgs84 %>%

pivot_longer(

cols = starts_with("psi_"),

names_to = "season",

values_to = "psi"

) %>%

mutate(season = ifelse(season == "psi_wet", "Wet Season", "Dry Season"))

# Plot site-level occupancy maps

ggplot() +

geom_sf(data = boundary, fill = NA, color = "black", size = 1) +

geom_sf( data = occ_map_long, aes(color = psi), alpha = 0.8, size = 4 ) +

scale_color_gradientn( colors = c("#FFE5B4", "#A6DDA3", "#2E7D32"), limits = c(0, 1) ) +

facet_wrap(~season) + labs(color = "Occupancy (ψ)", x = "Longitude", y = "Latitude") +

theme_minimal()

# END OF SCRIPT
